# Motivation and experiences of individuals with opioid use disorder and chronic pain using medical cannabis for 12 months

**DOI:** 10.1186/s12954-025-01306-9

**Published:** 2025-10-03

**Authors:** Michelle R. Lent, Karen L. Dugosh, Kartik Varsani, Andrea Jonsson, Tyler Kung, Katherine E. Galluzzi

**Affiliations:** 1 Department of Clinical Psychology, Philadelphia College of Osteopathic Medicine, 4190 City Avenue, Rowland Hall, Philadelphia, PA 19131 USA; 2 Research and Evaluation Group, Public Health Management Corporation, 1500 Market Street, Philadelphia, PA USA; 3 Philadelphia College of Osteopathic Medicine, 4190 City Avenue, Philadelphia, PA USA; 4 Department of Geriatric and Palliative Medicine, Philadelphia College of Osteopathic Medicine, 4190 City Avenue, Rowland Hall, Philadelphia, PA USA

**Keywords:** Medical cannabis, Opioid use disorder, Chronic pain, Qualitative research

## Abstract

**Background:**

The objective of this qualitative study was to better understand the experiences of individuals living with opioid use disorder (OUD) and chronic pain using medical cannabis (MC) for 12 months.

**Methods:**

Perspectives were captured via 10 key informant interviews conducted after 12 months of treatment with MC

**Results:**

Key themes identified regarding the reasons for initially pursuing MC included: (1) cannabis supply safety; (2) a desire to reduce or eliminate prescription medication usage for pain, OUD and mood; (3) to induce feelings of calm or relaxation; and (4) to improve levels of chronic pain. At 12 months, key themes identified surrounding their lived experiences with MC use were: (1) reductions in pain levels; (2) positive changes in emotional regulation and mood; (3) improvements in sleep quality and duration; and (4) reductions in cravings to use illicit drugs. The primary concerns expressed by interviewees surrounding MC use at 12 months related to changes in weight and appetite, and the cost of MC products.

**Conclusions:**

These qualitative data provide targets for future quantitative investigations of the potential mechanisms by which MC can impact recovery in the context of OUD.

## Introduction

FDA-approved agonist and partial agonist medications like methadone and buprenorphine, as well as the receptor antagonist extended-release naltrexone, are considered the standard of care for the treatment of opioid use disorder (OUD) [1, 2]. Results from observational studies and clinical trials showed significant reductions in drug-related deaths in those taking these medications for OUD (MOUDs) [3]. Despite the availability of these lifesaving medications, unintentional drug overdose continues to be a leading cause of accidental death in the United States [4, 5].

In recent years, researchers have studied the role that cannabis may play as a potential factor mitigating against opioid overdose deaths. Reductions in opioid overdose mortality rates have been observed in states in which cannabis was available for medical or recreational use [6]; a 2018 review of epidemiologic studies suggests that medical cannabis (MC) may reduce opioid use and harms [7]. Pritchett et al. [8] found MC use to be associated with reductions in patients’ opioid use without negative effects on function and therefore suggested that MC may provide an alternative pain medication option, and that some patients may reduce or cease the use of MOUD upon obtaining access to medical cannabis [8]. Further, MC and cannabinoids use may lessen overdose risk by reducing withdrawal symptoms and pain levels, two common antecedents to illicit opioid use [9, 10]. Despite the potential benefits of MC in OUD, the use of cannabis as adjunctive treatment to MOUD remains controversial [9]. Limited use of MC for OUD is likely fueled, at least in part, by the stigmatizing belief that use of MC in OUD is akin to substituting one drug for another. Additionally, while several randomized controlled trials suggest MC to be of benefit for the treatment of pain [10], the lack of randomized controlled trials evaluating the efficacy and safety of MC for OUD specifically likely also contribute to the limited adoption of this treatment approach.

In Pennsylvania (PA), MC is approved for the treatment of 24 qualifying conditions with the recommendation of a physician, including severe or intractable pain and OUD in combination with primary therapeutic interventions. The purpose of this qualitative study was to examine the motivations and experiences of adults taking MOUD and living with chronic pain who were initiating MC treatment for the first time. Data were collected via key informant interviews to identify the reasons why individuals receiving buprenorphine treatment for OUD who have co-morbid chronic pain initially seek MC treatment and what their experience has been after 1 year of MC treatment.

## Methods

Participants were previously enrolled in an observational, longitudinal pilot study of changes associated with MC treatment among individuals receiving MOUD who have comorbid chronic pain*.* Individuals aged 18 years or older were eligible to participate in the larger study if they had a first-time recommendation from a PA certifying physician for MC for OUD but had not yet started using MC, were taking buprenorphine for OUD (sublingual buprenorphine/naloxone or extended release buprenorphine), and reported a minimum pain score of 5 (out of 10) on the following question of the Brief Pain Inventory—Short Form: “Please rate your pain by marking the box beside the number that best describes your pain on the average” [11]. Individuals with prior recreational cannabis use were not excluded. Individuals who could not complete the consent process in English or who were not permitted to use MC (e.g., violates terms of probation/parole, against recovery housing rules) were excluded. Interested individuals met with an onsite research assistant to complete the informed consent process that included granting the research team access to their MC dispensary and medical records. Consenting participants then completed a baseline assessment that included demographic questions, measures of psychosocial functioning, and a urine drug screen, and they returned for study visits every 3 months for 1-year post-study entry. Further, we conducted retrospective chart review to obtain additional clinical data. Participants were offered a discount at a local MC dispensary a 1:1 tetrahydrocannabinol:cannabidiol (THC:CBD) 5 mg:5 mg oral capsule (30-day supply for $1 USD); however, participants were permitted to purchase any MC product in addition to the capsules. Participants were remunerated $30 USD for each study visit. Data were managed using REDCap (Research Electronic Data Capture) [12].

At month 12, research staff conducted key informant interviews about participants’ experiences using MC (interview guide, Appendix A) with the first 10 consenting participants from the larger trial (i.e., approximately 20% of the 47 enrolled participants). This sample size target was selected to capture a range of perspectives on MC use and was considered to be sufficient to approach thematic saturation, given that the narrow focus of the research question and homogeneity of the sample (i.e., all have OUD, are receiving buprenorphine MOUD treatment, and have chronic pain diagnoses [13, 14]). Interviewees were remunerated an additional $70. Participants provided verbal consent to be interviewed and for the interview to be audio recorded. Audio recordings were then transcribed with identifying information removed, and the audio recordings were then deleted. Interviews were conducted from February to September 2024, and generally lasted 30–40 min. The lead organization’s Institutional Review Board (#H18-054) approved the protocol and provided ethical oversight.

### Analyses

Descriptive statistics characterized the sample (e.g., means, standard deviations, frequencies) using IBM SPSS Statistics for Windows, Version 28.0 (Armonk, NY: IBM Corp). Qualitative analyses were informed by specific techniques of Grounded Theory [15, 16]. Specifically, techniques from the constant comparative method [16] were used to tally explicitly coded data on potential reasons for MC initiation and experiences with MC use. Data were inspected and all data coded before harm reduction-centered categories surrounding initiation (e.g., safety of supply, legal acquisition of MC) and 12 months of MC use (e.g., reductions in potential triggers for illicit opioid use such as pain, cravings, adverse emotions and sleep disturbances), consistently emerged. Coded incidents were compared and sorted further into the emergent categories [16]. We then hypothesized that individuals with OUD and chronic pain use or seek to use MC to minimize (1) known antecedents to illicit opioid use; and (2) risks associated with purchasing and/or using illicit cannabis.

## Results

Sample characteristics are in Table 1. The majority of participants completing key informant interviews were male (*n* = 6, 60%) and not Hispanic (*n* = 7, 70%) with a mean age of 44.9 years (*SD* = 10.6). The baseline pain score for key informant interviewees was 7.3 (*SD* = 1.6) and 90% (*n* = 9) reported a history of recreational cannabis use. At month 12, 90% had urinalysis-confirmed use of cannabis, suggesting that the vast majority of participants were actively using MC at the time of interview.

**Table 1:** Sample characteristics of individuals with opioid use disorder and chronic pain using medical cannabis for 12 months (*N* = 10)

	Mean (SD)	n (%)
Age (years)	44.9 (10.6)	–
Sex at birth		
Male	–	6 (60%)
Female		4 (40%)
Race		
Black	–	5 (50%)
White		3 (30%)
Other/missing		2 (20%)
Hispanic/latino		3 (30%)
Pain score at baseline (0–10)*	7.3 (1.6)	–
Employment status		
Full time		4 (40%)
Part time		0 (0%)
Unemployed		5 (50%)
Disability		1 (10%)

*Brief Pain Inventory- SF, Question #5

Key informant interviews (*n* = 10). Thematic analysis of the interview transcripts revealed three overarching themes: (1) the reasons for pursuing MC treatment; (2) experiences associated with using MC in the context of recovery from OUD; and 3) concerns or unintended outcomes of MC use (see Table 2).

**Table 2:** Key informant interview themes in individuals with OUD and chronic pain (*N* = 10)

*Reasons for MC initiation*
Safety
Reduce pain
Reduce or eliminate prescription medication use
Improve anxiety/relaxation and/or sleep
Prevent overdose/death
Reduce cravings for opioids
*Experiences using MC after 12 months*
Reductions in pain
Relaxation/calm/focus
Improved emotional regulation, mood/depression, irritability
Improved sleep quality and/or duration
Reductions in cravings for opioids/illicit drugs
Reductions in anxiety or panic, PTSD symptoms, stress
Reductions in use of MOUD and/or other prescription medications
*Concerns and unexpected outcomes of MC use after 12 months*
Weight or appetite change
Cost of physician recommender evaluations and/or dispensary MC products
Dry mouth
Headache
Non-opioid substance use
Legality/occupational
Adverse mood

1. *Reasons for initiating MC treatment for the first time.* Participants discussed several reasons for pursuing MC treatment, including:
  - *Safety*. Six participants reported a desire to cease purchasing cannabis “on the street,” and to utilize products that were free from potential contaminants, particularly fentanyl. One participant (Black female, age 52) stated, “Now these days I don't trust they [sic] weed because they putting fentanyl…and all that stuff like that. So, I feel safe at the marijuana dispensary.”
  - *Pain reduction.* Four participants discussed experiencing pain in their legs, back, and knees prior to MC use, and one noted pain to be the reason they began using (and ultimately misusing) opioids. One participant (Black male, age 57) discussed the analgesic impact of MC for their chronic back pain, “I was just trying to get anything to help… I went once and got a shot in my back but you know the shot they give you only lasted two days and I was right back to having pain, so that didn’t do no good. So, what the marijuana helps me, you know, it blocks out the pain, not all of it, but halfway, so that I can manage.”
  - *To reduce or eliminate prescription medication use to treat mood disorders, pain or OUD*. Four participants expressed a desire to decrease their use of prescription medication as reasons for initiating MC. “So, hopefully with the marijuana program and the marijuana that I have, I will be able to come off that medication [buprenorphine] as well. I’ve been on it for a long time as well too” (Black female, age 52).
  - *For anxiety reduction and relaxation*. Four participants discussed a desire to start using MC to enhance relaxation. One participant (Black male, age 43) reported, “I always hear weed was bad, bad, bad, bad, bad until, they come up with, oh, oh, weed help you with anxiety, will help calm you down.”

In summary, participants were motivated to initiate MC to assist in their OUD recovery to reduce or eliminate potential antecedents to illicit opioid use (negative affect, poorly managed pain) as well as the risks associated with purchasing cannabis illicitly, including the unintended use of fentanyl via the contaminated illicit cannabis supply.

2. *Experiences associated with MC use in the context of OUD recovery and/or pain treatment.* Several subthemes emerged surrounding participants’ experiences using MC for 1 year.
  - *Pain reduction*. All 10 participants reported experiencing reductions in pain that they associated with MC use. For example (Black male, age 57), “As far as the pain, they said it is getting worse. It [MC] helps like for me to be able to move around and help my mobile status because when I get up in the morning now, it is hard for me to get out the bed. So, the marijuana, it helps me make it through the day.” Another (White female, age 41) stated, “Since I got on the medical marijuana, I have been able to deal with my pain, and I have been in a lot of pain since I have came [sic] off the [illicit] opioids… so it has been really nice having to not worry about taking a prescribed medication [for pain]… or even the non-narcotic pain medications daily or mental health meds.”
  - *Improved emotional regulation and mood.* Five participants expressed predominantly positive changes in their anxiety severity (e.g., enhanced relaxation, feelings of calm, the ability to more effectively cope with stress, and better focus), as well as less irritability and depression, which were helpful in the context of their OUD recovery. One participant (White female, age 24) noted, “When you are getting like your body detox…you are very like irritable, like you get irritated so easily. Like for anything just, just for moving one thing that you want in one space to another… And it [MC] helped me out, go easily just like, going down slowly and not just like, go ahead like what I felt in the moment.” Similarly, another participant (Black male, age 48) said MC “helped with my anxiety and dealing with not using… it calms you down and it helps you with other ways especially when you are trying to get off other drugs.”
  - *Better sleep quality and duration*. Nine participants mentioned that their sleep quality and/or duration improved after starting MC treatment. One participant (Hispanic male, age 50) said, “I can sleep like a baby now.”
  - *Fewer cravings*. Six participants reported that for them, using MC was associated with fewer drug cravings. As one participant (Black male, age 57) stated, “I’m saying it [MC] reduced the cravings of other drugs, you know what I mean? It made my mind and body relaxed so I didn’t really think about it.”

Overall, participants endorsed that they felt MC assisted with their OUD recovery by improving potential triggers to illicit opioid use, including cravings to use substances, negative affect, pain, and sleep disturbances.

3. *Concerns and unexpected outcomes of MC use.* After the first 12 months of MC use, three participants noted experiencing changes in weight and appetite (both increases and decreases); for example, “It [MC] helps my stomach and gives me an appetite” (Black male, age 57). Additionally, approximately half of the participants discussed the high cost of dispensary MC products; for example, “If you want to get some good sh-t, you’re gonna’ spend some money, but the cost was a little high” (Hispanic male, age 50). However, some alternatively noted that cost was not a concern; for example, “The cost is a little high, but today everything is high” (Black male, age 48).

Generally, participants reported unexpected changes in their weight and appetite, as well as concerns regarding pricing, as negative aspects of the MC use after 12 months. Future studies could explore these concerns in more detail to better inform consent when initiating MC in the context of OUD.

## Discussion

This study is among the first qualitative investigations of the perceptions and experiences of new MC users who are currently engaged in buprenorphine for OUD and have comorbid chronic pain. Several notable themes emerged that may inform clinical patient-provider decisions about whether to incorporate MC into recovery treatment planning for members of this patient population. Additionally, these findings may help decision makers develop evidence-based policies surrounding the medicinal use of cannabis in the context of OUD, which remains a subject of significant scrutiny and debate [9].

Several participants indicated that they initiated MC treatment to legally obtain cannabis products that were free from the dangers of potential contaminants found in the illicit cannabis supply, including fentanyl. Exposure to illicit fentanyl is a primary driver of the current overdose epidemic [17] and therefore utilizing cannabis products that are manufactured by regulated grower/processors and dispensers could be viewed as a potentially life-saving harm-reduction strategy [18]. Participants also reported starting MC with the goal of reducing their use of certain prescription medications, including MOUD, analgesics, and anxiolytics, that they associated with negative side effects or safety risks. Many of our participants were already using cannabis illicitly and discussed the myriad of risks associated with purchasing cannabis off the street. Therefore, patients with OUD and their providers may benefit from discussing ways to obtain access to MC in states that allow for medicinal use to avoid legal risk and unintended consumption of other substances such as fentanyl, which could result in overdose or death. Further, MC products span a large variety of routes of administration and potency. Harm reduction discussions to MC use should also focus on selecting the safest routes of MC administration for each individual, the risks associated with cannabis exposure in adolescents and in pregnancy, and warning signs of potential adverse effects of use (e.g., cannabis hyperemesis syndrome, cannabis use disorder, exacerbation of certain mental health issues) [19].

Ineffectively managed pain is common in individuals in recovery from OUD [20], which can be an antecedent to illicit substance use. However, study participants consistently indicated that they experienced significant pain reduction with MC use; therefore, the analgesic potential of MC may be one mechanism by which MC has the potential to help prevent relapse. Further, participants reported that MC positively impacted the duration and quality of their sleep, as well as relatedly, their ability to relax or remain calm. These reports are consistent with findings from a systematic review of 39 studies that found modest improvement in sleep quality among individuals with chronic pain [21] and with a recent observational study of individuals with anxiety disorders that found significant reductions in anxiety severity after 3 months of MC use [22]. Improvements in pain, sleep, relaxation and mood also represent important potential functional mechanisms by which MC may promote sustained recovery in OUD. Given that participants commonly endorsed disturbances in sleep and mood prior to MC use, more intensive screening and monitoring of these concerns in individuals with OUD generally may be warranted. However, our study included a small sample size of individuals from only one U.S. state, limiting the generalizability of our findings, and included only individuals using buprenorphine-based MOUD. Additionally, most participants were using cannabis recreationally at baseline, which may have impacted their expectations surrounding MC use. Finally, we recruited participants at any stage of MOUD use (e.g., newly inducted, stable), which may confound the study findings. While differences in OUD or pain outcomes between MC and recreational cannabis user are not yet well-understood, the guidance and support of a physician, consistency of strain and potency, and lack of potential contamination from adulterants such as fentanyl distinguishes MC from recreational cannabis that may result in different clinical outcomes.

## Conclusions

As the OUD crisis continues to evolve, it is critical that adjunctive treatments to MOUD are identified and available that can address the range of biopsychosocial impairments that individuals with OUD and chronic pain experience. Understanding the perspectives and experiences of MC patients receiving MOUD and living with pain represents a first step to understanding the role MC may play in this regard. Future research using rigorous longitudinal designs is necessary to better understand how MC may impact recovery from OUD and chronic pain.

## Data Availability

No datasets were generated or analysed during the current study.

## Appendix 1

### Key informant interview guide

1. Could you please tell me a little about your experience with opioid use disorder and chronic pain?
2. Could you please talk a little about what first interested you? (prompts: *What made you interested in getting medical marijuana treatment? How were you hoping the medical marijuana could help you? What aspects of the program did you find most useful?).*
3. Tell me a little about the products you have used since obtaining your medical marijuana card.
4. Please talk a little bit about how the medical marijuana helped you to better manage your opioid use disorder. (prompts: *cravings, drug use, engagement with buprenorphine*?).
5. Tell me a little bit about how it helped you manage your chronic pain and associated problems. (prompts: *Changes in pain levels; mood; sleep*).
6. What were the downsides of medical marijuana treatment? (prompts: *Side effects? Access or cost?).*
7. Suppose you met someone who getting buprenorphine treatment for an opioid use disorder who also had chronic pain. If they asked you for your advice regarding medical marijuana as a treatment option, what would you tell them?
8. Is there anything else you would like to share with us about your experience with medical marijuana?

## Abbreviations

OUD: Opioid use disorder
MOUD: Medication for opioid use disorder
MC: Medical cannabis
